# The anti-leukemia activity and mechanisms of shikonin: a mini review

**DOI:** 10.3389/fphar.2023.1271252

**Published:** 2023-11-02

**Authors:** Han Dong, Chun-Di Chang, Fei Gao, Na Zhang, Xing-Jian Yan, Xue Wu, Yue-Hui Wang

**Affiliations:** ^1^ Department of Geriatrics, Jilin Geriatrics Clinical Research Center, The First Hospital of Jilin University, Changchun, China; ^2^ Department of Neurology, Jilin Province People’s Hospital, Changchun, China; ^3^ Endocrine Department, Qian Wei Hospital of Jilin Province, Changchun, China; ^4^ Electrodiagnosis Department, Jilin Province FAW General Hospital, Changchun, China; ^5^ Department of Urology, The First Hospital of Jilin University, Changchun, China

**Keywords:** leukemia, shikonin, anti-leukemia activity, mechanisms, multiple targets

## Abstract

Leukemia encompasses a group of highly heterogeneous diseases that pose a serious threat to human health. The long-term outcome of patients with leukemia still needs to be improved and new effective therapeutic strategies continue to be an unmet clinical need. Shikonin (SHK) is a naphthoquinone derivative that shows multiple biological function includes anti-tumor, anti-inflammatory, and anti-allergic effects. Numerous studies have reported the anti-leukemia activity of SHK during the last 3 decades and there are studies showing that SHK is particularly effective towards various leukemia cells compared to solid tumors. In this review, we will discuss the anti-leukemia effect of SHK and summarize the underlying mechanisms. Therefore, SHK may be a promising agent to be developed as an anti-leukemia drug.

## 1 Introduction

Currently, leukemia is still a group of life-threatening diseases, and ranks the sixth and eighth leading causes of death in male and female patients who suffer from cancers, respectively (Siegel et al., 2023). Leukemia is a series of malignant disorders that originate from clonal hematopoietic stem-progenitor cells which result in accumulation of abundant blasts in the bone marrow and extramedullary organs, and the normal hematopoietic system is seriously affected (Whiteley et al., 2021). Mechanistically, chromosomal alterations, genetic mutations, epigenetic dysregulation, metabolic abnormalities, and microenvironmental factors all serve critical roles in leukemogenesis that contribute to excessive proliferation, differentiation blockade, and resistance to apoptosis of leukemia cells (Brady et al., 2022; Yan et al., 2022; Egan and Schimmer, 2023). Furthermore, multiple intracellular signaling pathways are aberrantly activated in leukemia cells which are also involved in the pathogenesis of leukemia (Carter et al., 2020). In terms of classification, leukemia includes acute and chronic leukemia; the former consists of acute myeloid leukemia (AML) and acute lymphoblastic leukemia (ALL), and the latter is comprised of chronic myeloid leukemia (CML) and chronic lymphoblastic leukemia (CLL). With the emergence of tyrosine kinase inhibitor, CD20 monoclonal antibody, and inhibitors of Bruton tyrosine kinase, the prognoses of patients with chronic leukemia were substantively improved (Kantarjian et al., 2022; Langerbeins et al., 2022; Mauro et al., 2023). However, drug resistance is inevitable in many patients (Amarante-Mendes et al., 2022). Furthermore, the outcome of patients with acute leukemia is still dismal although new molecule targeted drugs, allogeneic stem cell transplantation, and immunotherapy are integrated into the overall treatment plan (Xu et al., 2019; Kreidieh et al., 2022). Thus, exploiting new treatment strategies is urgently needed in clinical practice.

In the past few years, phytochemicals have been widely used in the field of chemoprevention and cancer treatment as new therapeutic regimens, since plenty of the existing anticancer drugs are extracted from natural plants (Ranjan et al., 2019; Khatoon et al., 2022). Although many cytotoxic phytochemicals have been reported in a large number of literatures, only a few have anti-cancer activity *in vivo*, and their mechanism of action remain to be explored. Shikonin (SHK) is a naphthoquinone compound extracted from the root of traditional Chinese medicine *Arnebia euchroma* or *Lithospermum erythrorhizon*, which has antiviral, antibacterial and anti-inflammatory effects and can be widely used in medical, agricultural, and animal husbandry fields (Guo et al., 2019). In addition, SHK also has good coloring and is commonly used in pharmaceutical additives, cosmetics, food processing, and other industries (Guo et al., 2019). In the last 3 decades, the anti-cancer effect of SHK and its derivatives at the cellular and molecular levels have been investigated in detail through *in vitro* and *in vivo* experiments (Boulos et al., 2019). The synergistic effect of SHK in combination with existing chemotherapeutic drugs, immunotherapy, and other treatments further reinforces the potential of this phytochemical to be integrated into standard treatment regimens (Tabari et al., 2022; Tang et al., 2023). The anti-cancer effect and its underlying mechanisms of SHK were well reviewed by other investigators (Boulos et al., 2019; Yan et al., 2023). Moreover, there are studies showing that SHK is particularly effective towards various leukemia cells compared to solid tumors (Wiench et al., 2012; Wiench et al., 2013). Thus, this review is focused on the anti-leukemia activity of SHK and its mechanisms.

## 2 The anti-leukemia effect of SHK and its underlying mechanisms

**FIGURE 1:**
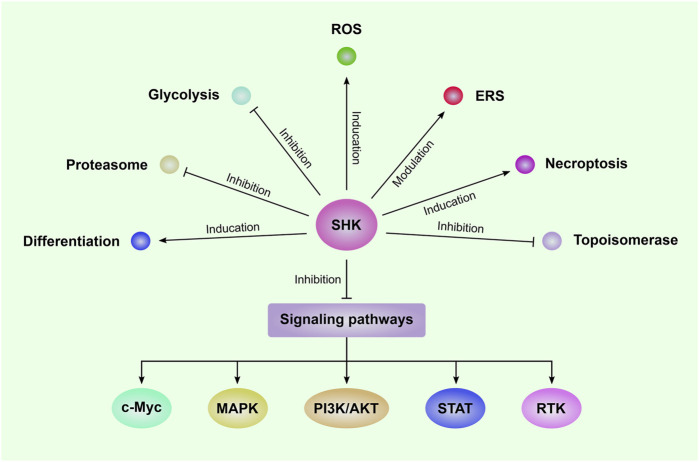
The mechanisms of SHK-mediated anti-leukemia effect Shikonin (SHK) can inhibit the proliferation and induce apoptosis in a series of leukemia cells. The underlying mechanisms include induction of reactive oxygen species (ROS) generation and necroptosis; inhibition of glycolysis, proteasome, topoisomerase, and several signaling pathways; modulation of endoplasmic reticulum stress (ERS); promote leukemia cell differentiation. MAPK: mitogen-activated protein kinase; PI3K/AKT: phosphatidylinositol 3-kinase/protein kinase B; STAT: Signal transducer and activator of transcription; RTK: receptor tyrosine kinase.

The anti-leukemia effect of SHK was firstly reported by Yoon and colleagues in 1999, in which SHK was found to induce death of HL-60 human promyelocytic leukemia cell line via inducing apoptosis as shown by increased DNA fragmentation and percentage of hypodiploid cells companied with activation of caspase-3 (Yoon et al., 1999). Since then, SHK was widely investigated by numerous studies as an anti-leukemia agent (Hsu et al., 2004; Mao et al., 2008; Chen et al., 2012). Regulation of cell cycle progression also plays an important role in the anti-leukemia effect of SHK (Thangapazham et al., 2008; Wiench et al., 2012; Shan et al., 2017). The cell cycle of NB4 cells was arrested in G phase after treated with SHK (Shan et al., 2017). SHK was reported to cause an arrest of U937 cells in G1 and S phase and to decrease expression of cell cycle-related proteins, such as cyclin D, cyclin-dependent kinases (CDK), and proliferating cell nuclear antigen (PCNA) (Thangapazham et al., 2008; Wiench et al., 2012). Moreover, SHK showed the activities to regulate drug resistance of leukemia cells (Huang et al., 2020). SHK could overcome resistance of CML cells to tyrosine kinase inhibitors (TKI) by downregulation of miR-92a-1-5p, a poor-prognosis marker frequently overexpressed in patients with leukemia (Huang et al., 2020). The mechanisms of the anti-leukemia effect mediated by SHK include inducing reactive oxygen species (ROS) generation, modulating glucose metabolism, and inhibiting multiple signaling pathways which will be described in detail below and a summary of these mechanisms was presented in Figure 1.

### 2.1 Induction of ROS generation

ROS are increasingly recognized as regulators of cellular signaling, and keeping ROS levels low is essential to both hematopoietic stem cells and leukemia stem cells (Romo-González et al., 2022). Inducing the generation of ROS is a pivotal mechanism for apoptosis of leukemia cells (Sumi et al., 2022). Several studies have demonstrated that SHK could inhibit the proliferation and induce apoptosis of leukemia cells via ROS generation (Mao et al., 2008; Duan et al., 2014; Boonnate et al., 2023). Treatment of K562 CML cells with SHK results in profound induction of apoptosis accompanied with rapid generation of ROS, marked release of the mitochondrial proteins cytochrome c and Smac/DIABLO, and activation of caspase-9 and -3, which could be completely blocked by scavenging of ROS (Mao et al., 2008). In a recent study, SHK was reported to induce apoptosis of adult T cell leukemia/lymphoma cells via generation of ROS, loss of mitochondrial membrane potential and induction of endoplasmic reticulum stress (ERS). Similarly, a ROS scavenger, N-acetylcysteine, could prevent apoptosis and reverse both loss of mitochondrial membrane potential and ERS (Boonnate et al., 2023). These studies indicate that SHK-induced oxidative injury operates at a proximal point in the apoptotic signaling cascades, and subsequently activates the stress-related pathways and leads to apoptosis of leukemia cells (Mao et al., 2008; Boonnate et al., 2023). The mechanism of inducing ROS generation by SHK was studied in HL-60 leukemia cells. Cytosolic thioredoxin reductase is an important selenocysteine (Sec)-containing antioxidant enzyme. SHK primarily targets the Sec residual in cytosolic thioredoxin reductase to inhibit its physiological function, and shifts the enzyme to an NADPH oxidase to generate superoxide anions, leading to accumulation of ROS (Duan et al., 2014). Collectively, SHK could promote ROS generation by targeting antioxidant enzyme to induce apoptosis of leukemia cells and ROS is an upstream trigger of SHK-induced apoptosis.

### 2.2 Modulation of glucose metabolism

Exploiting metabolic vulnerability of leukemia cells is a novel strategy for treatment of patients with leukemia. Leukemia cells prefer aerobic glycolysis over oxidative phosphorylation (Qing et al., 2021). Upregulation of genes involved in glycolysis and concomitant downregulation of tricarboxylic acid genes were observed in B precursor ALL lymphoblasts compared with normal hematopoietic progenitor cells, while inhibition of glycolysis induces the apoptosis of leukemia cells, suggesting a dependence on glycolysis for leukemia cell survival (Boag et al., 2006). The ETS-related transcriptional factor Fli-1 plays an important role in the induction and progression of leukemia in part by inducing glycolysis (Sheng et al., 2022). As a pyruvate kinase M2 (PKM2) inhibitor, SHK could inhibit the proliferation of HEL leukemia cells through inhibition of glycolysis. Furthermore, downregulation the expression of Fli-1 in a dose-dependent manner by SHK indicating it may suppress glycolysis indirectly (Sheng et al., 2022). C-MYC and GLUT2 are two key regulators of glycolysis, which are targets of Hippo signaling pathway in human Jurkat leukemia cells (Vališ et al., 2016). SHK could inhibit lactate production and Jurkat cell proliferation by activation of Hippo signaling pathway, accompanied with downregulation of C-MYC and GLUT2 expression (Vališ et al., 2016). Thus, inhibition of glycolysis is an important mechanism for SHK to function its anti-leukemia effect.

### 2.3 Inhibition of multiple signaling pathways

During the development and progression of leukemia, multiple cell proliferation and anti-apoptotic signaling pathways are activated to sustain the rapid proliferation. Targeting these aberrant pathways are potential strategies for the treatment of leukemia (Pan et al., 2017; Stone et al., 2017; Shanafelt et al., 2022). SHK was reported to have the ability to inhibit multiple signaling pathways in leukemia cells (Shan et al., 2017; Chen et al., 2018; Todorovic et al., 2021). In a network pharmacology study, the mechanism of action of the anti-leukemia effect mediated by components from *A. euchroma* was explored, and several apoptosis and inflammation-related biological signaling pathways were identified, such as mitogen-activated protein kinase (MAPK) signaling, phosphatidylinositol 3-kinase (PI3K)/protein kinase B (AKT) signaling, IL-17 signaling, and T cell receptor signaling pathways (Wang et al., 2020). These pathways are related to inhibiting survival and inducing apoptosis of leukemia cells, relieving inflammatory environment, and inhibiting angiogenesis (Wang et al., 2020).

#### 2.3.1 PI3K/AKT

Deregulations of PI3K/AKT pathway are frequently associated with cancerogenesis, especially in a wide range of hematological malignancies (Gao et al., 2016). SHK was reported to exert its antitumor activity in Burkitt’s lymphoma by inhibiting PI3K/AKT/mTOR pathway (Ni et al., 2018). Wiench et al. reported that integration of bioinformatics and three “-omics” assays demonstrated that SHK affected the PI3K/AKT pathways in leukemia cells, and a decrease in phosphorylated AKT was observed after SHK treatment (Wiench et al., 2013). In U937 leukemia cells, inhibiting phosphorylation of AKT after SHK treatment could be confirmed as well (Zhao et al., 2015). SHK could suppress the proliferation and migration of CML K562 leukemia cells and induce apoptosis by increasing PTEN level and inactivated PI3K/AKT signaling pathway (Chen et al., 2018). Interestingly, the ubiquitin ligase Cbl proteins may negatively regulate PI3K/AKT signaling pathway in SHK-induced apoptosis (Qu et al., 2015). Hence, inhibition of PI3K/AKT signaling pathway is one important mechanism for the anti-leukemia activity of SHK.

#### 2.3.2 MAPK

The MAPK pathway is a common point of convergence of many different mitogenic and anti-apoptotic signal transduction pathways in hematological malignancies (Chakraborty et al., 2021), which includes extracellular signal-regulated kinase (ERK), p38, and c-Jun NH(2)-terminal kinase (JNK), with each MAPK signaling pathway consisting of at least three components, a MAPK kinase kinase (MAP3K), a MAPK kinase (MAP2K), and a MAPK (Peterson et al., 2022). SHK could inhibit the proliferation and induce apoptosis of acute promyelocytic leukemia NB4 cells companied with an increase in phosphorylation of p38 MAPK and JNK, while the expression of phosphorylated ERK was decreased (Shan et al., 2017). Furthermore, inhibiting the phosphorylation of ERK1/2 is an important mechanism for SHK to kill U937 leukemia cells (Zhao et al., 2015). Thus, regulation of MAPK signaling pathway plays a critical role for SHK to exert its biological function.

#### 2.3.3 c-Myc

c-Myc is very important in tumorigenesis and the potential mechanisms may include enhancing cell proliferation, inhibition of cell death, modulating metabolism, promoting angiogenesis, and regulating stem cell formation (Duffy et al., 2021). Downregulation of c-Myc expression by SHK in leukemia cells has been reported in several studies (Zhao et al., 2015; Vališ et al., 2016; Shan et al., 2017). SHK and its derivatives showed potent anti-leukemia effect, and downregulation of c-Myc was identified as the most commonly mechanism in a series of leukemia cell lines (U937, Jurkat, Molt4, CCRF-CEM, and multidrug-resistant CEM/ADR5000) (Zhao et al., 2015). Molecular docking studies revealed that shikonin and its derivatives bind to the same DNA-binding domain of c-MYC as the known c-MYC inhibitors (Shan et al., 2017). Inhibiting c-Myc expression by SHK was also validated in separate studies with Jurkat and NB4 leukemia cells (Vališ et al., 2016; Shan et al., 2017). Therefore, inhibition of c-Myc expression is a novel mechanism to explain the anti-leukemia effect of SHK.

#### 2.3.4 Signal transducer and activator of transcription (STAT)

Numerous studies have confirmed the association between activating mutations in JAK-STAT and hematologic disorders (Fasouli and Katsantoni, 2021). SHK derivatives, isobutyrylshikonin and α-methylbutyrylshikonin, show potent activity against CLL and B-cell prolymphocytic leukemia cells by triggering apoptosis, inhibiting cell proliferation, and attenuating leukemia cell stemness, and decrease in expression of phosphorylated STAT3 and its downstream-regulated molecules is the major mechanism (Todorovic et al., 2021). These results highlight the necessity of further testing of SHK derivatives as possible new anti-leukemia agents or auxiliary drugs via inactivating the STAT signaling pathway.

#### 2.3.5 Receptor tyrosine kinase

Substantial evidence has shown that deregulation of the receptor tyrosine kinase is important for maintaining the survival of malignant cells of hematopoietic origin (Vishwamitra et al., 2017; Daver et al., 2021; Katagiri et al., 2022). Type I insulin-like growth factor receptor (IGFIR) and its primary ligand IGF-I play critical roles in the development and progression of leukemia (Vishwamitra et al., 2017). A dose-dependent inhibition of IGFIR kinase activity by SHK was observed using a radiometric protein kinase activity assay with a IC50 concentration of 2.6 μM, indicating a direct inhibitory effect of IGFIR by SHK (Wiench et al., 2013). Beta-hydroxyisovalerylshikonin, a SHK derivative, could inhibit the protein tyrosine kinase activities of epidermal growth factor receptor (EGFR) and v-Src, and the mechanism of inhibition is competitive with respect to the peptide substrate (Nakaya and Miyasaka, 2003). Accordingly, SHK may be a potential inhibitor of receptor tyrosine kinase to exert additional anti-leukemia activities.

### 2.4 Induction of necroptosis

Emerging studies demonstrate that necroptosis plays a crucial role in both solid tumors and leukemia (Gong et al., 2019; Fang et al., 2023). It was reported that necroptosis related signaling pathways were continuously activated in leukemia cells, and inhibition of these pathways could be an alternative treatment strategy for AML patients (Xin et al., 2017). Necroptosis-related gene signature was adopted to establish a risk stratification system in patients with AML (Fang et al., 2023). SHK is studied as a necroptosis inducer in a wide range of hematological and solid neoplasms (Wada et al., 2015; Huang et al., 2020; Markowitsch et al., 2021; Wang et al., 2022b). Apoptotic resistance to TKI targeting the BCR/ABL fusion protein represents a major challenge in the treatment of CML, and induction of nonapoptotic cell death is a valuable strategy in this respect. SHK could overcome TKI resistance in CML cells by inducing necroptosis via activation of receptor-interacting protein kinase 1 (RIPK1)/RIPK3/mixed lineage kinase domain-like protein (MLKL) signaling both *in vitro* and *in vivo* (Huang et al., 2020). However, inhibition of necroptosis could enhance SHK-induced apoptosis of leukemia cells also could be observed. Necroptosis inhibitor necrostatin-1 targeting RIPK1 or siRNA-mediated knockdown of RIPK1 significantly enhanced SHK-induced apoptosis in K562, HL-60, and primary leukemia cells (Han et al., 2012). The underlying mechanisms appear to be associated with the inhibition of RIPK1-dependent phosphorylation of ERK1/2 (Han et al., 2012). These results indicate that SHK could induce different forms of programmed cell death in leukemia cells, and apoptosis and necroptosis may be mutually exclusive in SHK-induced death.

### 2.5 Modulation of endoplasmic reticulum stress (ERS)

ERS and activation of unfold protein response are the major factors contribute to chemoresistance in cancer cells (Xia et al., 2021). Induction of ERS is an important mechanism for SHK-induced apoptosis in prostate cancer cells, 5-fluorouracil-resistant colorectal cancer cells, and colon cancer cells (Gara et al., 2015; Han et al., 2019; Piao et al., 2022). In adult T cell leukemia/lymphoma cells, activation of ERS was related to SHK-induced cell apoptosis (Boonnate et al., 2023). However, cell proteome analysis demonstrated that SHK could significantly downregulate ERS protein ERP57 expression after HL-60 treated with SHK (Trivedi et al., 2016). Knockdown of ERP57 expression increases SHK-induced apoptosis, whereas overexpression of ERP57 results in protection of HL-60 cells from apoptosis (Trivedi et al., 2016). Similarly, ERS compromised the cytotoxicity of SHK against glioblastoma stem cells (GSC), and inhibiting ERS with 4-phenylbutyric acid markedly enhanced the cytotoxicity of SHK in GSC (Liu et al., 2015). Thus, the contradictory roles of ERS in SHK-induced death of leukemia cells may suggest the anti-leukemia effect of SHK shows leukemia type specificity.

### 2.6 Inhibition of proteasome

Proteasome inhibition has been demonstrated to be a potential treatment strategy in patients with leukemia (Csizmar et al., 2016; Kamens et al., 2023). Pre-clinical studies have showed that proteasome inhibition impedes proliferative cell signaling pathways and exhibits cytotoxic synergism with other chemotherapeutics agents (Csizmar et al., 2016; Roeten et al., 2021). Furthermore, clinical trials incorporating bortezomib plus chemotherapy regimens have reported a range of responses in AML patients, with complete remission rates >80% in some cases (Csizmar et al., 2016). Inhibition of proteasome activity participates in the anti-tumor and anti-inflammatory effects of SHK (Wada et al., 2015; Yan et al., 2015). Murine P388 leukemia cells and hepatoma H22 cells, and human prostate cancer PC-3 cells were induced to death by SHK via inhibiting the proteasome activity followed by accumulation of ubiquitinated proteins and several proteasome target proapoptotic proteins (Yang et al., 2009). The carbonyl carbons C(1) and C(4) of SHK potentially interact with the catalytic site of beta 5 chymotryptic subunit of the proteasome by computational modeling prediction (Yang et al., 2009). Thus, inhibition of the proteasome activity by SHK may contribute to its anti-leukemia property.

### 2.7 Inhibition of DNA topoisomerase

Topoisomerases are essential enzymes that modulate DNA under- and overwinding, knotting, and tangling (Pendleton et al., 2014). Moreover, they are also the targets for some of the most widely used anticancer drugs against leukemia, such as etoposide and mitoxantrone (Pendleton et al., 2014). As early as 1995, SHK was reported as an inhibitor of DNA topoisomerase-I (Ahn et al., 1995), and SHK could inhibit growth and induce apoptosis of GSC and glioma cells by inhibiting topoisomerase-I (Zhang et al., 2013). SHK and its derivatives as potent anti-cancer agents target against topoisomerases is summarized by a recent review (Olatunde et al., 2023). SH-7 is a new naphtoquinone compound derivative of SHK exhibiting significantly inhibitory actions on topoisomerase I/II. SH-7 showed marked apoptosis-inducing function on HL-60 leukemia cells, which was validated to be of mitochondria-dependence (Yang et al., 2006). Accordingly, SHK may be a potential drug to inhibit topoisomerases to exert its anti-leukemia effect.

### 2.8 Promotion of leukemia cell differentiation

Induction of leukemia cell differentiation has achieved milestone success in acute promyelocytic leukemia (APL) with the use of arsenic trioxide and all-trans retinoic acid (Wang et al., 2022a; Kutny et al., 2022). SHK was also reported to be an inducer of cell differentiation in human HL-60 APL cells in two previous studies (Zhang et al., 2012; Guo et al., 2022). HL-60 cells treated with a submicromolar concentrations of SHK, and a strong dose-response relationship between SHK exposure and the features of differentiation was showed in terms of morphology changes, nitroblue tetrazolium reductive activity, and the expression levels of surface antigen CD11b/CD14 (Zhang et al., 2012). Further mechanism research showed that activation of Nrf2/ARE pathway modulates the intercellular redox homeostasis towards oxidation was necessary to support SHK-induced differentiation (Zhang et al., 2012). Additionally, SHK at the non-cytotoxic concentration could decrease Wilms’ tumor 1 (WT1) protein and simultaneously reduce CD34 protein and increase the CD11b protein expression in a dose-dependent manner in HL-60 cells (Guo et al., 2022). Hence, modulation of intercellular redox homeostasis and downregulation of WT1 expression both are associated with differentiation of HL-60 leukemia cells, and SHK may be an agent for leukemia differentiation therapy under non-cytotoxic concentration.

### 2.9 Synergistic anti-leukemia effect with other drugs

The synergistic effects between chemotherapeutic agents, antibiotics and SHK were widely studied (Wang et al., 2019; Li et al., 2022; Tabari et al., 2022). Due to the different mechanisms of inhibiting receptor tyrosine kinase, SHK derivative beta-hydroxyisovalerylshikonin showed synergistic effect with imatinib on the induction of apoptosis in K562 cells (Nakaya and Miyasaka, 2003). A high-throughput screening approach was utilized to explore a library of natural products to determine the most synergistic combination in precursor-B cell ALL (Sweeney et al., 2020). Finally, dimethylaminoparthenolide and SHK were identified to effectively inhibit proliferation resulting in cell death in primary and immortalized leukemia cells (Sweeney et al., 2020). In addition, dimethylaminoparthenolide and SHK have been shown separately to inhibit cell survival and proliferative signaling and activate tumor suppressors and proapoptotic pathways (Sweeney et al., 2020). Therefore, SHK may be incorporated into current regimens to treat patients with leukemia.

## 3 Summary and prospectives

During the past 30 years, the anti-leukemia effect of SHK is widely investigated against different types of leukemia cells. Nevertheless, it is still unknown what type of leukemia cells is the most sensitive to SHK. There are now substantial research results showing that the mechanisms of anti-leukemia activities mediated by SHK include induction of ROS generation and necroptosis, modulation of glycose metabolism and ERS, and inhibition of proteasome, DNA topoisomerases and multiple signaling pathways. Furthermore, SHK may be an inducer for leukemia cell differentiation. These results suggest that SHK can target multiple molecules to exert its biological function. Consequently, unlike many drugs used in clinic target relative a single molecule, this highlights the special advantage of SHK for avoiding drug resistance compared with single target agents during treatment. However, it should be cautious to interpret the results of the anti-leukemia mechanisms of SHK as limited evidence is reported in some aspects and contradictory results exist. Moreover, further studies that can elucidate the mechanism of action more concisely need to be performed since many studies just showed relatively superficial mechanisms. Currently, there is only one clinical trial reporting that SHK was used to treat 19 patients with late-stage lung cancer, in which inhibition of tumor growth and improved immune functions were observed (Guo et al., 1991). Furthermore, the quality of life of the patients was substantially improved as showed by alleviated chest pain and cough, while the appetite and body wight loss were improved. Additionally, no obviously adverse side-effect was found (Guo et al., 1991). However, more evidence on the efficacy and safety of SHK in the treatment of patients with leukemia or other cancers is still needed. There are other factors also should be considered before development of SHK as an anti-leukemia drug. On one hand, SHK is a water-insoluble component, and exploration of optimal drug dosage form is necessary for its clinical usage. In recent years, nano drug delivery systems have emerged as promising strategies to improve the bioavailability and enhance the therapeutic efficacy of SHK and various drug delivery systems were developed which are expected to tested in clinical setting (Long et al., 2023; Shi et al., 2023; Yan et al., 2023). On the other hand, a combination between SHK and new treatment strategies used in recent years is limited. Several studies have already reported that SHK showed immunomodulatory effects, such as downregulation of PD-L1 expression (Yuan et al., 2023) and improvement of immune organ damage mediated by tumors (Long et al., 2012). Hence, the efficacy of SHK combining with immune checkpoint inhibitors are anticipated to evaluate. Finally, the toxicity of SHK may require reevaluation *in vivo* as we observe obvious ascites in mice after intraperitoneal injection. However, no significant toxicity was observed in rats treated with SHK derivatives for a long-term administration period by gavage (Su et al., 2013), which indicates the toxicity of SHK may be associated with different drug delivery routes. In summary, the current research results demonstrate that SHK is a promising anti-leukemia agent that may be used in clinic although some issues should be addressed in order to take advantage of its efficacy and simultaneously decrease its potential side effects.
